# A Novel Approach for Tuning the Physicochemical, Textural, and Sensory Characteristics of Plant-Based Meat Analogs with Different Levels of Methylcellulose Concentration

**DOI:** 10.3390/foods10030560

**Published:** 2021-03-08

**Authors:** Allah Bakhsh, Se-Jin Lee, Eun-Yeong Lee, Nahar Sabikun, Young-Hwa Hwang, Seon-Tea Joo

**Affiliations:** 1 Division of Applied Life Science (BK21 Four), Gyeongsang National University, Jinju 52852, Korea; drallahbakhsh1@yahoo.com (A.B.); sejinlee1994@gmail.com (S.-J.L.); ley9604@gmail.com (E.-Y.L.); seturajjo@yahoo.com (N.S.); 2 Institute of Agriculture & Life Science, Gyeongsang National University, Jinju 52852, Korea; philoria@hanmail.com

**Keywords:** plant-based meat analog, commercial texture vegetable protein, texture soy isolate protein, methylcellulose

## Abstract

This study assessed the effects of Methylcellulose (MC) at different concentrations on plant-based meat analog (PBMA) patties, comprised of commercial texture vegetable protein (C-TVP) and textured isolate soy protein (T-ISP) as key ingredients, and compared to beef patty control. A significantly higher difference was observed in moisture content in control with increasing MC concentration than the C-TVP and T-ISP patties. However, protein varied significantly among three different protein sources, with control had higher protein content than PBMA patties. Crude fiber content recorded higher values in C-TVP as compared to control. Significantly lower pH values were recorded in control than C-TVP and T-ISP respectively. Regardless, with the addition of MC or ingredient PBMA and control patties tend to reduce lightness (L*) and redness (a*) value after cooking. Although control sample before cooking exhibits lighter and redder than PBMA patties (C-TVP and T-ISP). Likewise, water holding capacity (WHC) decreases as the concentration of MC increases (1.5–4%) in control and PBMA patties. Warner-Bratzler shear force (WBSF) and texture profile analysis (TPA), including hardness, chewiness, and gumminess of control, were significantly higher than C-TVP and T-ISP. Consequently, panelists’ in the sensory analysis presented that C-TVP patties containing 3% of MC had better sensory properties than T-ISP. Hence, PBMA patties with C-TVP and incorporation of 3% MC are considered ideal for manufacturing of meat analog as related to control (beef).

## 1. Introduction

The term “meat analog” denotes food products that are not made from red meat exclusively, commonly known as meat alternatives, meat substitutes, fake, mock, and imitation meat [1]. However, it possesses texture, mouth-feel, taste, and nutritional qualities that resemble meat [2]. Meat contributes to the food industry by supplying specific functionalities and has its attraction on consumers for its organoleptic features. Meat proteins are responsible for their characteristic appearance, textural and functional properties [3]. However, mimicking these meat protein characteristics by any other source of protein is difficult. Moreover, recently the International Agency for Research on Cancer, the cancer agency of WHO (World Health Organization), has classified the consumption of red meat (particularly processed meat) as carcinogenic to humans [4]. Furthermore, Food and Agricultural Organization (FAO) reports have been critical of the ecological impact of high levels of meat consumption and potentially transmissible diseases [5,6]. To mask these disadvantages of red meat, meat analogs are just one example of a variety of products recently demanded by a substantial portion of the population, especially those concerned about red meat’s potential health effects [7]. Additionally, the projections for the increasing demand for animal protein in the coming decades are distressing, while extensive livestock production is also causing severe environmental and ecological imbalance [8]. Consequently, the research community is targeting refining the current production systems, searching for efficient novel technologies, while at the same time focusing on the improvement of consumption habits and food cultures [9]. 

In the current study, soy-based texture vegetable protein (TVP), and textured isolate soy protein (T-ISP) have been used as a meat replacer with many economic and functional benefits [3]. Soy-based TVP_S_ are plant-based protein products with low saturated fat, a high concentration of essential amino acids, and is cholesterol-free [10]. The manufacturing process of TVP involves a high-pressure extrusion process and a final spinning or extraction of the finishing product, which can then be used in meat analogs [7]. The low/intermediate moisture TVP has advantages in handling, storage, and shelf stability but requires time to hydrate before consumption. Upon hydration, it presents a spongy textured, fibrous structure mimicking meat [7]. Furthermore, numerous investigators have reported that by using soy protein and wheat gluten as TVP constituents, the final product could mimic the texture, appearance, taste, smell, and functionality of red meat [5]. 

In red meat, textural and taste parameters are important to the consumers and represent high economic value as some cuts bring exorbitant prices. In contrast, meat analogs lack these features and are generally regarded as substandard to cheaper meats. Numerous plant proteins, including cereal, oilseed, legume, and soy proteins (textured, flour, concentrate, and isolate), are recommended additions to the meat analogs. These elements have appropriate functional properties (e.g., water and oil absorption capacity, emulsification), which allow them to create numbers of distinctive meat substitutes [5,11].

The binding ability of the different ingredients in plant-based meat is of significant importance as non–adhesive behavior of varying plant ingredients can significantly affect the final analogy. Earlier binding agents such as egg solids, hydrocolloids, starch, and milk protein have been used in various commercial products [12]. In the present study, Methylcellulose (MC) has been used as a binder. Quality characteristics of MC include binding abilities and moisture retention, boil-out control, increase volume, and texture improvement in several types of meat analogs and processed meat [13]. Through synthetic modification, the naturally occurring polymer cellulose is converted to hypromellose or MC and is considered safe for consumption by humans [13]. Moreover, MC is classified as GRAS (generally recognized as safe) by the FDA (21 CFR 182.1480) and is also allowed in USDA regulated meat patties at concentrations up to 0.15% (9 CFR 3 t 8.7). Previously, the use of binding agents in meat analogs has been widely investigated, although no such attempt has been made to study the effects of MC on quality characteristics of Plant-based meat analog (PBMA) patties. Therefore, the objective of the present study was to evaluate the effects of MC on quality characteristics of PBMA with the incorporation of different texturized soy vegetable proteins.

## 2. Materials and Methods

### 2.1. Materials

Commercial texture vegetable protein (C-TVP) (Anthony’s goods, Glendale, CA, USA) and ISP (isolate soy protein) (Shandong, China) were as the base for PBMA and MC (high viscosity, Modernist Pantry, Eliot, ME, USA) was incorporated as a binder. Other ingredients, including molasses, yeast seasoning, umami seasoning, coconut oil, canola oil, garlic powder, and pepper were used in the formulation (Table 1). 

### 2.2. Sample Preparation and Processing

**Figure 1:**
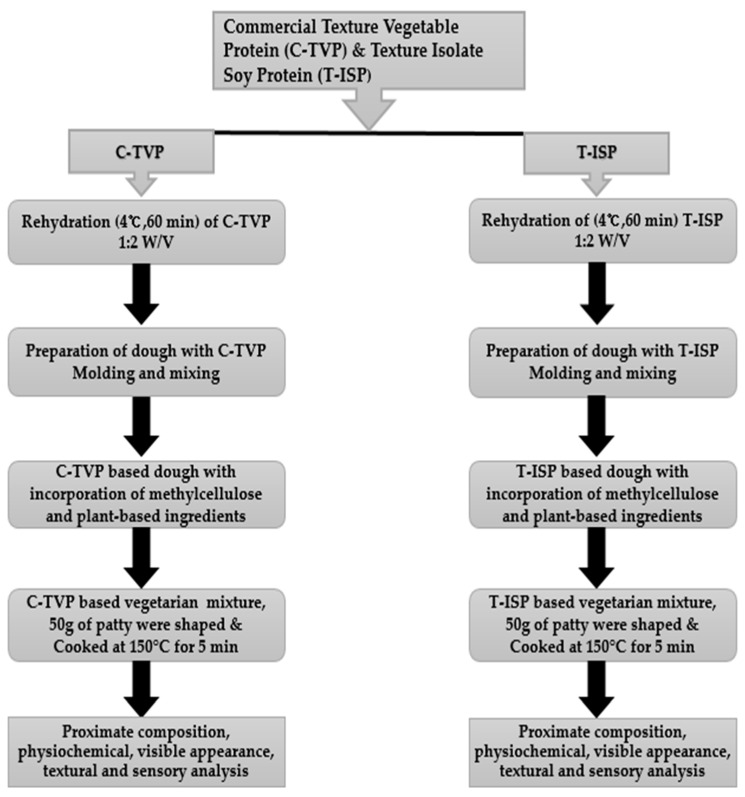
Flow diagram for manufacturing the meat analog. C-TVP: Commercial textured vegetable protein. T-ISP: Textured isolate soy protein. PBMA: Plant-based meat analog.

The flow diagram for processing meat analog is described in Figure 1. For meatless patties, C-TVP and texture isolate soy protein (T-ISP) were used as the base. The texturization of ISP was carried out by mixing ISP powder with water at a ratio of 1:6 (*w*/*v*). The mixture was stirred continuously over a lower flame until it forms a thickened paste. Subsequently, the paste was heated in an oven for two hours at a temperature of 120 °C T-ISP was a secondary option for comparing the quality characteristics of the created meatless patties to C-TVP. A total of three hundred g of each C-TVP and T-ISP were mixed with water separately (2 times in volume) and allowed to hydrate for “1 h” at 4 °C for a single concentration of MC with three repetitions and two formulations having raw and cooked patties respectively. After that, the hydrated C-TVP and T-ISP were mixed with the ingredients listed in Table 1 using a Kitchen Aid (Classic Plus Stand Mixer, St Joseph, MI, USA). Subsequently from the whole mixture, 50 g of the mixture was then shaped into patties using a patty press maker. The current experiment had three different concentrations of MC (1.5%, 3%, and 4%), from every single concentration of MC three patties (repetition) were prepared with one control and two treatments. In total, for one control, two treatments, and two formulations, 27 raw and 27 cooked patties were prepared. Therefore, in total, 54 patties were shaped. Eighteen patties were allocated for each control and two treatments separately.

A beef patty was used for the control formulated as describe in Table 1. The patties were cooked by dry heat, cooking on a non-stick pan at 150 °C for 5 min per side. They were flipped three times or until the internal temperature reached 75 °C as measured by a probe thermometer. Patties were allowed to cool at ambient temperature for 30 min before measuring the physicochemical and sensory attributes.

### 2.3. Proximate Analysis

Moisture, protein, fat, and ash contents were determined based on the standard AOAC [14]. Moisture content was quantified by the oven (BioFree, BF-150C, Buchen Korea), drying 5 g samples at 105 °C for 16 h. Protein was determined by the established procedure of Kjeldahl assay N analyzer (B-324, 412, 435 and 719 S Titrino, BUCHI, Flawil, Switzerland) (N × 6.25) using 0.1 g of sample. The crude protein was determined by using the following formula.

(1) $$\%N=\frac{\left[V\left(1\right)-V\left(B1\right)\right].F.c.F.M\left(N\right)\times 100}{M.1000}$$

% P = % N × PF(2)

V(1): consumption of titrant, sample (mL)

V(BI): average consumption of titrant, blank (mL)

F: molar reaction factor (1 = HCl, 2 = H_2_SO_4_)

c: concentration of titrant [mol/L]

M(N): molecular weight of N (14,007 (g/mol))

M: sample weight (g)

1000: conversion factor (mL in L)

PF: protein factor 

% N: % of weight of N

% P: % of weight of protein

Crude fat was measured with 2 g samples by extraction in a Soxhlet apparatus (MS-EAM9203-06, Seoul Korea) by using petroleum ether as a solvent. The crude fat content was calculated by using the following formula.

(3) $$\%\mathrm{Crude}\mathrm{fat}=\left(W2-W1\right)\times \frac{100}{S}$$

Weight of empty flask (g) = W1

Weight of flask and extracted fat (g) = W2 

Weight of sample = S

Ash was determined after incineration of 2 g of sample in a furnace (CFMD2, Changsin, Korea) at 500 °C. Crude fiber determination was estimated using an Ankom 200 Fiber Analyzer (Ankom Technology, Macedon, NY, USA) by digesting 0.5 g with H_2_SO_4_ and NaOH. The loss of weight resulting from ashing (2 h at 600 ± 15 °C) was collected to calculate the crude fiber content [15]. 

### 2.4. Physicochemical Analysis

The pH values of raw and cooked patties were measured with a digital pH meter (Mettler Toledo, MP230, Schwerzenbach, Switzerland) using 3 g of sample homogenized with 20 mL of distilled water. 

The color of raw and cooked patties was measured using a Konica Minolta Colorimeter (Chroma meter, CR-300, Japan). The apparatus was standardized through a white ceramic plate (Y = 93.5, X = 0.3132, y = 0.3198), and lightness (L*), redness (a*), and yellowness (b*) values were recorded.

Release water percentage (RW%) was measured based on a method described by Joo [16]. The cooking loss (CL%) was determined as a percentage method adopted by Biswas et al. [17] using the following formula: Cooking loss (%) = (Weight of the patties after cooking/Weight of the patties before cooking) × 100. 

Warner-Bratzler shear force (WBSF) was determined on the cooked sample using the established AMSA procedure [18]. The shrinkage percentage of the patties’ diameter was measured at four different locations both before and after cooking. A total of 18 (nine raw and nine cooked) patties were allocated for physiochemical analysis.

### 2.5. Visible Appearance 

The appearance of the control and PBMA patties were assessed by adding the different concentrations of MC (1.5%, 3%, and 4%) respectively. The external and internal appearance were photographed using a digital camera (EOS 700D, Canon, Tokyo, Japan), and various features were distinguished. In total, 18 (nine raw and nine cooked) patties were used for visible appearance.

### 2.6. Texture Profile Analysis

Samples were uniformly cut into 1 × 1 × 1 cm, and they were axially compressed using a Sun Rheometer (Compact-100 II, Sun Scientific Co., LTD., Tokyo, Japan) with a flat pressure adaptor of 25 mm in diameter (No. 1). The samples were compressed at a crosshead speed of 60 mm/min at a final strain of 60% through a 2-cycle sequence with a load cell of 10 kg [19]. The following parameters were determined: hardness, cohesiveness, springiness, gumminess, and chewiness. A total of nine patties were assigned for the determination of texture profile analysis.

### 2.7. Sensory Evaluation

A 10-member trained panel from the laboratory of meat science Gyeongsang National University Korea, with 20 members of the untrained panel, includes students and researchers from the Department of Animal Sciences at Gyeongsang National University, Republic of Korea, assessed sensory characteristics of prepared patties. The panelist assortment was approved according to Lawless and Heymann [20], modified by Rahman et al. [21]. Small pieces of different samples (2 cm × 2 cm × 2 cm) were prepared and marked, random coding was allotted on pre-positioned glass container (Pyrex, Charleroi, PA, USA), and the pieces of samples were permitted to rest for 30 min at room temperature and then disseminated among the panelists. For judging each sample in a triplicates way, fluorescent light was used. For every sensory evaluation procedure, the panelist was provided with drinking water for washing the mouth for every new sample evaluation. Sensory traits that were recorded included appearance, shape, firmness, color, and overall acceptability. The samples were judged using a 9-point hedonic scale ranging from extreme dislike (score = 1) to extreme like (score = 9). A total nine number of PBMA patties were assigned for sensory evaluation.

### 2.8. Statistical Analysis

The results of PBMA based on C-TVP and T-ISP content are represented as the mean plus/minus standard error of the mean (SEM). The effect of main ingredients and concentration of MC on the variation of proximate composition, physicochemical properties, and visible appearance was described as mean and standard error of mean (SEM). Analysis of variance (factorial ANOVA) was carried out using SPSS version 23 (IBM Corp., Armonk, NY, USA). For multiple mean comparisons, the Tukey’s test was run at the level of 5%.

## 3. Results and Discussion

### 3.1. Proximate Analysis

The proximate composition of control and PBMA patties are presented in Table 2. Moisture content prepared from a lower MC concentration (1.5%) was not statistically different among treatments. However, control beef patties with a higher concentration of MC (3–4%) expressing a higher moisture content than patties prepared from soy-based C-TVP and T-ISP. The ability of MC in reducing the loss of moisture content was due to the thermal gelation of MC. During heating, MC formed an adhesive layer, which acted as a barrier to prevent moisture loss [22]. 

The mechanism by which MC gelation is achieved between meat protein and plant-based protein is still unclear. One standard theory is that when in solution, hydrophobic methyl groups along the methylcellulose polymers are surrounded by cage-like structures of water molecules [23]. With increasing temperature, the cage structure is disrupted, and the polymers gradually lose their hydrated water. At the gelation point, polymers’ association occurs due to extensive hydrophobic associations between exposed hydrophobic segments [24]. Elevated temperatures highly favor the hydrophobic associations, and strong gels can form [25]. 

However, in the current study protein belongs to a heterogeneous mixture of different sources. Therefore, purifying the protein following different sources will result in different protein profiles, quality, and functionality [26]. The protein content of three types of patties’ varied significantly between various protein sources, with control (beef) indicated higher protein than PBMA patties. Although at any concentration of MC, there was no significant difference detected between C-TVP and T-ISP. 

Consequently, PBMA patties with different MC concentrations exhibit no major (*p* > 0.05) difference in fat content among control (beef), C-TVP, and T-ISP respectively. It has been reported that the fat content of plant-based meat is rationally varied as compared to traditional patties [27], however, the fat content of the present study was within the range of Bohrer [27]. Generally, meat analogs are considered low in fat and protein content; however, the new generation of meat analogs products contain substantially greater fat and protein content than traditional meat analog products [9]. Therefore, our argument regarding the average level of fat and protein in meat analog was supported by Ahirwar et al. [28] who described that ready-to-eat meat analog has a good percentage of protein and average fat content manufactured from vegetable and cereal sources. 

Irrespective with an application of different concentrations of MC or C-TVP and T-ISP ash content showed no difference. As expected, fiber content for PBMA patties was recorded higher than the control sample, with C-TVP represents the highest value. Similar results were also reported by Bohrer [27] in modern meat analogs. The higher fiber in PBMA patties was probably due to the plants and polysaccharides incorporated into the plant-based patties recipe. The fibrous nature of meat alternatives gives good textural and sensory sensation. Additionally, dietary fiber has been considered to play an essential role in preventing large bowel disease, ischaemic heart disease, and diabetes mellitus [29].

### 3.2. Physicochemical Analysis

The physiochemical indicators, including pH and colorimetric evaluation, are given in Table 3. There was a significant difference in pH between meat analogs and control (beef patties). The lower pH value of control was likely due to the regular glycolytic changes in meat [30]. However, C-TVP and T-ISP showed a pH of more than 6. The higher pH of PBMA could be due to the slight alkalinity of TVP (pH 7.42–7.43) [31]. Consistent with the current study, Bell and Shelef [32] recorded the pH of minced meat containing vegetable protein had higher pH than as compared to control, while Ahmad et al. [33] also determined that integration of soy protein isolate at 25% expressively increase the pH in meat sausage, which is similar to the outcomes of the present study. 

Likewise, pH and calorimetric measurements are interconnected with each other. The color coordinates are considered to be one of the essential physical properties in determining consumer acceptance of the product. All patties tended to decrease in lightness (L*) and redness (a*) after cooking. The results show that the control sample before cooking was lighter and redder than PBMA patties (C-TVP and T-ISP). However, our results were in contrast with the reported results of the literature on L* and a* values. Deliza et al. [34] reported an increase in the textured soy protein concentration in beef patties increased the L* values, but a* values were not statistically different. Hidayat et al. [35] also found a similar observation on the beef sausage. The variation of L* and a* values in the present study compared to other studies could be due to the substitution of plant-based proteins (100% substitution) in the formulation. The small globules from meat, such as water and fat, can cause more light reflection, which will probably contribute to higher lightness [36].

The a* values of control before cooking were higher than C-TVP and T-ISP treatments due to the myoglobin pigment in red meat. However, an increase in myoglobin denaturation can be shown by the lower a* values after applying the heat treatment [37], which in tandem with our result in Table 3. The cooking did not affect the redness values of textured soybean protein incorporated samples [34]. Similar effects were noticed in the raw and cooked samples incorporated with either C-TVP or T-ISP as described in Table 3. The b* values of C-TVP and T-ISP before cooking were higher than control. The yellowish coloration of PBMA patties can be associated with the yellow color of soy protein ingredients. Subsequently, the yellowish-brown color initially, affecting the final products’ [9]. However, MC’s concentration at different percentages only plays a minor role in reducing the b* values of raw and cooked patties.

In the current study, WHC is expressed in two parts, RW and CL, shown in Table 4. The concentration of MC had a significant effect on the RW and CL. An increase in MC concentration from 1.5% to 4.0% lowered the RW and CL in all treatments. These findings were similar to the result reported by Hill and Prusa [38] for beef patties. They described that cellulose hydrocolloids bind moisture in product formulation, and it can gel upon heating. According to Hill and Prusa [38], surface moisture probably would not be affected by gum addition; therefore, evaporative losses were not affected by treatment. Consequently, the present data shows that MC’s incorporation did not increase cooked moisture content, but it generally reduced total cook loss. Previously Arora et al. [3] proved that carrageenan and xanthan gum types binding agents had a higher yield than protein-based binding agents. At the same time, they concluded that WHC depends upon protein binding properties, which consequently agreed with our results. 

Subsequently, WBSF for control represents the highest value, and there is no significant difference between C-TVP and T-ISP treatments. The softer textural properties of C-TVP and T-ISP affect their shear force values. Ruiz de Huidobro et al. [39] reported that shear force value was significantly correlated to hardness, springiness, and chewiness. The shear force in meat is a good measure of initial bite tenderness, which can cause changes during the cooking process are related to heat-induced alteration of myofibrillar proteins and connective tissue, as solubilizes the connective tissue leading to meat tenderization. In contrast, the denaturation of myofibrillar proteins causes meat toughening [40]. The finding of the current study aligns with the Danowska-Oziewicz [41], who detected lower values shear force for the samples containing soy isolate protein as likened to control (pork patties). 

Diameter before and after cooking of control and PBMA patties are presented in Table 4. The degree of shrinkage (diameter after) was ranged from about 17.46–22.68% for control, 4.23–12.28% for C-TVP, and 3.64–8.98% for T-ISP. Control represents higher shrinkage due to the connective tissue denaturation and fluid (moisture and fat) loss Table 4. The substitution with plant-based protein reduces the shrinkage markedly in T-ISP, although no difference to C-TVP. According to Gujral et al. [42] the addition of fibers and non-meat protein ingredients may reduce diameter shrinkage and weight loss. Similarly, in the current study, the increase in MC concentration (4%) decreases shrinkage of all patties (control: 17.46%, TVP: 4.23%, and T-ISP: 3.64%).

### 3.3. Visible Appearance

**Figure 2:**
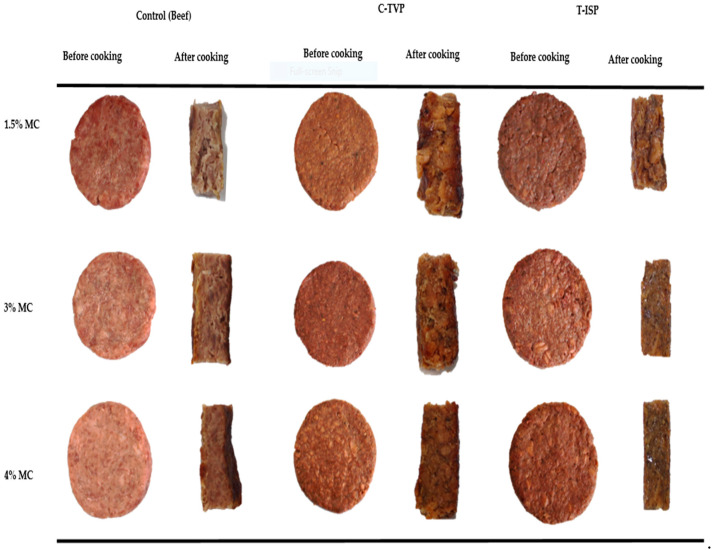
The external-internal appearance of cooked and uncooked plant-based meat patties.

The external and internal appearance of meat analogs before and after cooking has been presented in Figure 2. The external appearance before cooking showed no difference in observation at different concentrations of MC. However, MC’s effect can be seen after thermal treatment, in which the higher concentration (4%) can maintain the structure of patties. MC is essentially incorporated in some modern meat analog due to product consistency and binds all ingredients together to be more intact and stable [9]. MC is a useful binder, especially on the meat analog that does not require pre-heat for gel formation due to its unique thermal gelling and right emulsifier properties [43].

The drawback of using TVP and T-ISP is that we can see the patties’ surface’s granular appearance. The internal appearance of all patties appeared more homogenous and cohesive with a higher concentration of MC. This proved that the addition of MC could bind well all the ingredients. The interior of C-TVP and T-ISP patties show a rough with intact and no crack appearance. Nevertheless, the interior of C-TVP shows more finely structure than T-ISP patties. The probable reason could be due to adequate hydration of C-TVP during the preparation of the dough. Earlier, MC’s phenomena as a binder have been reported, which confirmed that MC helps maintain product shape and firm texture in various commercially available products, i.e., impossible burgers and beyond burgers [27].

### 3.4. Texture Profile Analysis

**Figure 3:**
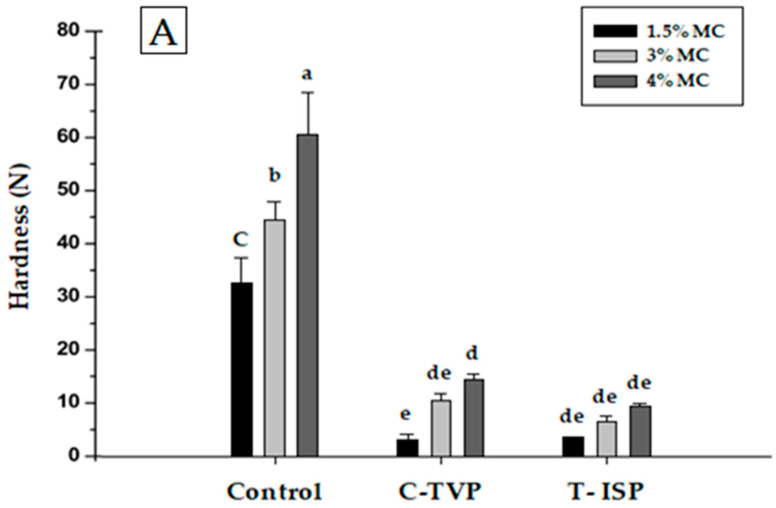

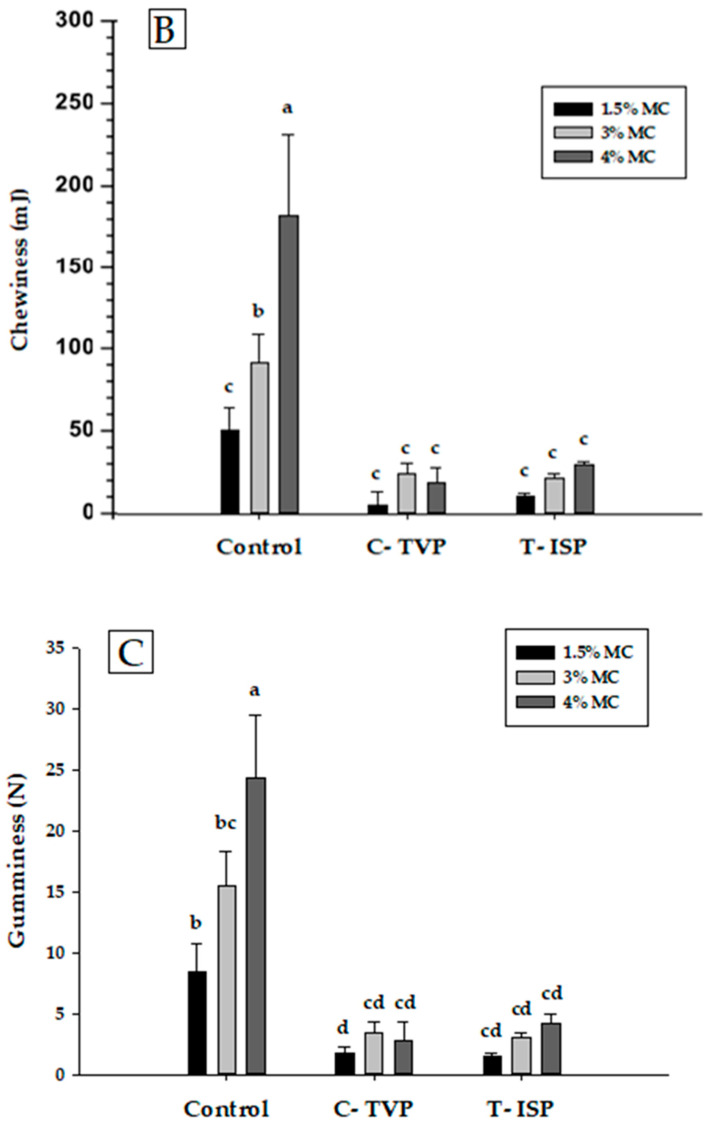

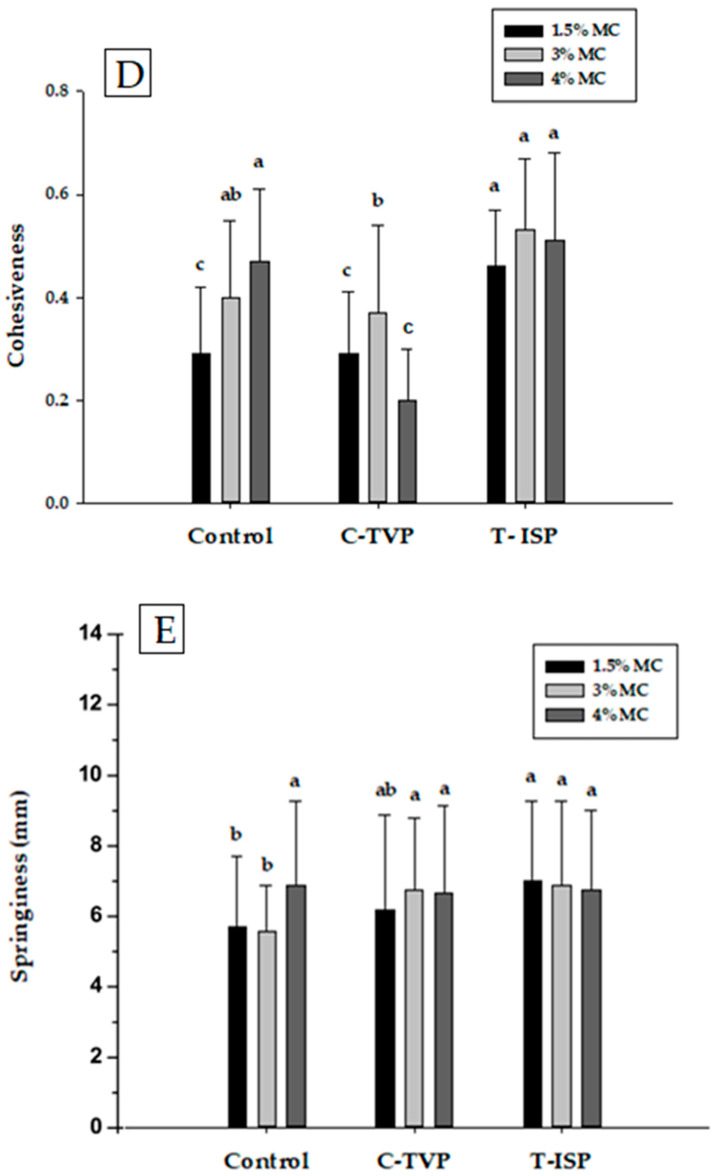
The texture profile analysis (TPA) parameters of plant-based meat on the type of texture soy isolate protein and different methylcellulose concentrations. (**A**) Hardness (N); (**B**) Chewiness (mJ); (**C**) Gumminess (N); (**D**) Cohesiveness; (**E**) Springiness (mm). Those are just different concentrations of MC (Methylcellulose) and the concentration is there on the top right.

The textural properties are crucial for developing meatless patties because in meat analog’s texture is an essential factor in mimicking the organoleptic taste of muscle. Figure 3 illustrates the textural parameters, including hardness, chewiness, gumminess cohesiveness, and meat analogs’ springiness with different concentrations of MC%. The hardness, chewiness, and gumminess of control were significantly higher in comparison to C-TVP and T-ISP. The higher hardness in control was expected due to the muscle proteins denaturation phenomenon, which led to hardness in the meat system [19]. It is evident from the shrinkage percentage shown in Table 4, whereby meat protein has a higher degree of shrinkage than plant-based proteins. An increase in MC concentration from 1.5% to 4% increases ‘hardness of all patties. The current result was consistent with the results reported by Arora et al. [3], who described that when the binding agent increased, the hardness, chewiness, gumminess, and compression values increased proportionally. Similarly, Ayadi et al. [44] reported that incorporating carrageenan at higher concentration (0.5% to 1.5%) increased hardness of sausage products. The reason for lower hardness values in TVP and T-ISP treatments were due to extensive hydration of textured protein with water at the early stage of the processing phase, ultimately causes the PBMA patties to be softer. According to, Ruiz de Huidobro et al. [39] hardness, chewiness, and springiness are instrumental parameters for assessing meat texture. 

However, in the present study, only springiness values of PBMA patties (TVP and T-ISP) showing marginally or no difference to the control. The hardness and chewiness values were showed a substantial difference between treatments and control. As we mentioned earlier (introduction), to mimic conventional beef patties’ textural properties is the most challenging part in the development of meat analogs. 

### 3.5. Sensory Evaluation

**Figure 4:**
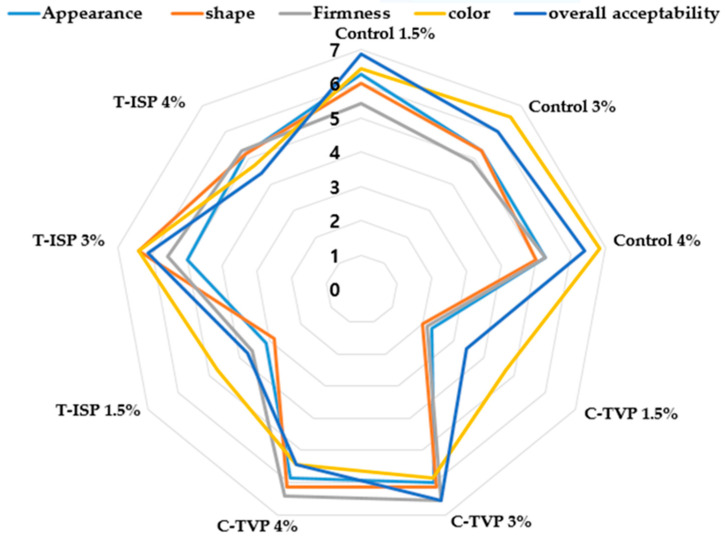
Sensory profile of plant-based meat-based with different soy isolate protein and methylcellulose percentage.

Sensory parameters are a chief concern for the development of PBMA patties using MC as a binder. The sensory traits for control (beef), C-TVP and T-ISP are presented in Figure 4. Based on the percentage of MC, the control patties expressing higher values in 4% MC for shape, firmness and color, although panelists scored higher, appearance and overall acceptability with 1.5% MC respectively. C-TVP patties obtained the highest score for appearance, shape, firmness, color and overall acceptability with 3% and 4% of MC concentration. Though, T-ISP samples incorporating 3% MC performed well than 1.5% and 4% MC concentration. The subjective evaluation demonstrated a clear preference towards 3% MC in PBMA patties (C-TV and T-ISP). Samples with the integration of 1.5 and 4% of MC were the least preferred on sensory evaluation basis. In contrast to our study, Imkyung et al. [45] described that with hydroxypropyl methylcellulose application as an animal fat replacer for meat patties, there is no significant difference in color, flavor and taste; however, tenderness, juiciness and overall acceptability show a statistically significant difference.

Based on previous literature, it has been reported that the application of water alone in ground beef patties in control without methylcellulose and hydroxypropyl methylcellulose did not satisfy the sensory panel; they further reported that color and aroma of ground patties were least affected by the application of methylcellulose [46]. The vast variability of PBMA patties as compared to control could be due to plant-derived proteins (soy and wheat protein) in meat analogs expressing more elastic, rubbery and chewy sensation and poor mouth feel due to their agglomeration properties. 

Moreover, previous literature confirmed that incorporating a different type of soy family (soy paste, soy protein isolate or texture soy protein) generates a unique beany essence in meat products and downgrade sensory scores [41]. Remarkably, in the current study, no beany essence was noticed. The possible reason might be due to various types of plant-based ingredients (Table 1) used to mask the beany flavor in PBMA patties successfully. Furthermore, due to natural differences between muscle and plant materials, i.e., structure and size of protein molecules, amino acid composition, peptide sequence, and the chemical composition of both intracellular and extracellular materials, it is difficult to reproduce the complex and delicate sensory profile of animal meat products.

## 4. Conclusions

The present study assessed the physicochemical, textural, and sensory properties of PBMA patties with two types of texturized soy isolate protein (C-TVP and T-ISP) and incorporation of different concentrations of binding agent (MC). The addition of MC significantly affected the quality characteristics of C-TVP and T-ISP-based PBMA patties. C-TVP with 3% MC showed promising results, with adequate physicochemical, textural parameters, and with satisfactory patty visible appearance, thereby improving the comprehensive process yield compared to T-ISP. Although samples with 4% MC also exhibit similar results compared to 3 % MC, they failed to satisfy the sensory panelist in C-TVP and T-ISP. Using beef as a control, it can be concluded that C-TVP with a 3% MC (binding agent) is recommended to prepare acceptable PBMA patties with good physicochemical, textural, and sensory acceptability.

## Figures and Tables

**Table 1:** Treatment and formulation of plant-based meat analogs.

Ingredients % Concentration (MC)				Treatments					
		Control (Beef)		C-TVP				T-ISP	
	1.5%	3%	4%	1.5%	3%	4%	1.5%	3%	4%
Lean beef C-TVP T-ISP	82.1	80.82	80.00	76.08	74.90	74.13	70.08	74.90	74.13
Methylcellulose	1.5	3.00	4.00	1.50	3.00	4.00	1.50	3.00	4.00
Garlic powder				2.28	2.25	2.22	2.28	2.25	2.22
Yeast extract				2.28	2.25	2.22	2.28	2.25	2.22
Black pepper				1.52	1.50	1.49	1.52	1.50	1.49
Mushroom				2.28	2.25	2.22	2.28	2.25	2.22
Salt				1.14	1.11	1.11	1.14	1.11	1.11
Beef back fat	16.4	16.18	16.00						
Coconut oil Canola oil				3.80 3.80	3.75 3.75	3.71 3.71	3.80 3.80	3.75 3.75	3.71 3.71
Beet juice				3.04	3.00	2.96	3.04	3.00	2.96
Molasses				1.52	1.50	1.49	1.52	1.50	1.49
Umami seasoning				0.76	0.74	0.74	0.76	0.74	0.74

C-TVP: Commercial textured vegetable protein. T-ISP: Textured isolate soy protein. MC: Methylcellulose.

**Table 2:** Proximate chemical composition of plant-based meat and control (beef) with different concentration of methylcellulose.

Ingredient	Beef (Control)			C-TVP			T-ISP			SEM	P Ingredient	P Concentration	P ing * P Conc.
Concentration	1.5%	3%	4%	1.5%	3%	4%	1.5%	3%	4%				
Moisture	57.49 ^b,c,d^	59.46 ^a,b^	62.64 ^a^	57.43 ^b,c,d^	51.54 ^e,f^	48.32 ^f^	57.80 ^b,c^	53.77 ^d,e^	54.28 ^c,d,e^	1.21	<0.001	0.026	<0.001
Protein	20.56 ^b^	21.19 ^b^	21.18 ^b^	16.48 ^b^	16.77 ^b^	16.93 ^b^	16.96 ^b^	16.95 ^b^	16.07 ^b^	0.71	0.079	0.650	0.345
Fat	18.38 ^a^	19.12 ^a^	18.85 ^a^	14.28 ^a^	15.05 ^a^	15.83 ^a^	17.25 ^a^	16.13 ^a^	15.28 ^a^	2.17	0.126	0.997	0.945
Ash	2.92 ^a,b^	2.22 ^b^	2.64 ^a,b^	3.11 ^a^	3.24 ^a^	2.84 ^a,b^	2.65 ^a,b^	2.83 ^a,b^	2.65 ^a,b^	0.25	<0.001	0.857	0.831
Crude fibre	1.38 ^c^	1.56 ^c^	1.69 ^c^	6.04a ^b^	6.87 ^a^	7.82 ^a^	3.15 ^c^	3.66 ^bc^	3.70 ^b,c^	0.90	<0.001	0.499	0.932

^a–f^ Different superscript letters within the same row mean significantly different between treatments (*p* < 0.05). SEM: standard error of mean; *: interaction between ingredient and concentration. C-TVP: Commercial texture vegetable protein; T-ISP: Texture isolate soy protein

**Table 3:** Physiochemical characteristics of plant-based meat and control (beef) with different concentration of methylcellulose.

Ingredient	Beef (Control)			C-TVP			T-ISP			SEM	P Ingredient	P Concentration	P ing * P Conc.
Concentration	1.5%	3%	4%	1.5%	3%	4%	1.5%	3%	4%				
pH before	5.54 ^d^	5.35 ^d^	5.52 ^d^	6.34 ^c^	6.50 ^b,c^	6.35 ^c^	6.28 ^c^	6.68 ^b^	7.08 ^a^	0.08	<0.001	0.002	<0.001
pH after	5.71 ^e^	5.63 ^e,f^	5.51 ^f^	6.43 ^c^	6.20 ^d^	6.77 ^a,b^	6.15 ^d^	6.61 ^b^	6.88 ^a^	0.05	<0.001	<0.001	<0.001
L before	46.47 ^a^	47.83 ^a^	46.93 ^a^	45.91 ^a^	39.95 ^b^	38.88 ^b,c^	34.70 ^d^	36.63 ^c,d^	40.16 ^b^	0.85	<0.001	0.456	<0.001
a before	17.83 ^a^	16.05 ^b^	16.25 ^b^	12.39 ^d^	14.09 ^c^	11.68 ^d,e^	12.94 ^c,d^	10.83 ^e,f^	9.90 ^f^	0.41	<0.001	<0.001	0.001
b before	11.87 ^c,d^	11.24 ^d^	11.55 ^d^	17.50 ^a^	13.37 ^b,c^	13.76 ^b^	12.04 ^c,d^	11.02 ^d^	11.85 ^c,d^	0.54	<0.001	0.001	0.011
L after	38.48 ^a^	35.46 ^a,b^	32.20 ^b,c,d^	29.15 ^d^	31.15 ^c,d^	30.45 ^c,d^	31.33 ^c,d^	30.71 ^c,d^	32.93 ^b,c^	1.10	<0.001	0.470	0.009
a after	8.59 ^b,c^	8.73 ^b,c^	7.75 ^c^	10.08 ^a,b^	9.77 ^a,b^	10.01 ^a,b^	10.62 ^a^	9.60 ^a,b^	8.52 ^b,c^	0.53	0.004	0.089	0.359
b after	11.36 ^a,b^	12.34 ^a,b^	10.19 ^a,b^	14.30 ^a,b^	12.89 ^a,b^	11.49 ^a,b^	15.61 ^a,b^	12.60 ^a,b^	15.56 ^a,b^	1.57	0.061	0.539	0.476

^a–f^ Different superscript letters within the same row mean significantly different between treatments (*p* < 0.05). SEM: standard error of mean; *: interaction between ingredient and concentration. C-TVP: Commercial texture vegetable protein; T-ISP: Texture isolate soy protein.

**Table 4:** Water-holding capacity and tenderness related measurement of plant-based meat and control (beef) with different concentration of methylcellulose.

Ingredient	Beef (Control)			C-TVP			T-ISP			SEM	P Ingredient	P Concentration	P ing * P Conc.
Concentration	1.5%	3%	4%	1.5%	3%	4%	1.5%	3%	4%				
Release water (%)	3.9 ^a^	1.8b ^c,d^	1.8 ^b,c,d^	4.06 ^a^	2.21 ^b,c^	1.44 ^c,d^	3.47 ^a^	2.43 ^b^	1.77 ^d^	0.20	0.980	<0.001	0.044
Cooking loss (%)	7.91 ^c,d^	6.55 ^d,e,f^	5.36 ^f^	9.98 ^b^	8.69 ^b,c^	7.12d ^e^	12.01 ^a^	7.30 ^c,d,e^	6.11 ^e,f^	0.48	<0.001	<0.001	0005
WBSF (N)	3.6 ^b,c^	3.80 ^b^	4.26 ^a^	2.14 ^f,g^	2.74 ^e^	3.20 ^d^	2.41 ^e,f^	2.41 ^e,f^	3.29 ^c,d^	0.12	<0.001	<0.001	0.044
Diameter before	14.99 ^a,b^	15.83 ^a,b^	15.52 ^a,b^	16.63 ^a,b^	15.47 ^a,b^	15.83 ^a,b^	16.30 ^a,b^	15.37 ^a,b^	15.70 ^a,b^	0.38	0.256	0.406	0.121
Diameter after	11.59 ^d^	12.30 ^c,d^	12.81 ^c,d^	14.96 ^a^	13.57 ^b,c^	15.16 ^a^	15.08 ^a^	14.29 ^a,b^	14.81 ^a,b^	0.41	<0.001	0.308	0.038

^a–f^ Different superscript letters within the same row mean significantly different between treatments (*p* < 0.05). SEM: standard error of mean; *: interaction between ingredient and concentration. C-TVP: Commercial texture vegetable protein; T-ISP: Texture isolate soy protein; WBSF: Warner-Bratzler shear force.
